# Varicella-zoster virus post-exposure management and prophylaxis: A review

**DOI:** 10.1016/j.pmedr.2019.101016

**Published:** 2019-11-06

**Authors:** Anne M. Lachiewicz, Megan L. Srinivas

**Affiliations:** a University of North Carolina, Division of Infectious Diseases, USA

**Keywords:** Varicella-zoster virus, Varicella, Herpes zoster, Immunocompromised, Vaccination, Post-exposure prophylaxis, Varicella zoster immune globulin, VARIZIG

## Abstract

Varicella-zoster virus causes both varicella (chickenpox) and herpes zoster (shingles). Although varicella incidence has dramatically declined since introduction of the live-attenuated varicella vaccine, vaccination rates are suboptimal, and outbreaks still occur. Additionally, herpes zoster incidence continues to rise. Severe or fatal complications may result from varicella transmission to at-risk individuals who are exposed to either varicella or herpes zoster. An increasing number of children and adults are receiving immunosuppressive therapies and are at high risk for severe varicella and other complications if exposed to the virus. Clinical management of individuals exposed to varicella-zoster virus should take into consideration the type of exposure, evidence of immunity, and host-immune status with regard to ability to receive varicella vaccination safely. Post-exposure varicella vaccination may prevent infection or mitigate disease severity in persons eligible for vaccination. Post-exposure prophylaxis with varicella zoster immune globulin is indicated for populations ineligible for vaccination, including immunocompromised children and adults, pregnant women, newborns of mothers with varicella shortly before or after delivery, and premature infants. Appropriate post-exposure management of individuals exposed to either varicella or herpes zoster—including assessment of immune status and rapid provision of optimal prophylaxis—can help avoid potentially devastating complications of varicella-zoster virus infection.

## Highlights

• Risk of primary varicella-zoster infection persists among children and adults.
• Severity risk is higher among immunocompromised, pregnant, and newborn hosts.
• Exposure, evidence of immunity, and immune status determine recommended prophylaxis.
• Vaccination may prevent infection or mitigate disease severity in eligible persons.
• Varicella zoster immune globulin is recommended for certain individuals.

## 1 Introduction

Varicella-zoster virus (VZV) causes 2 diseases: varicella (also known as chickenpox) and herpes zoster (HZ, also known as shingles). Primary infection with VZV causes varicella. After primary infection, VZV persists in a dormant state in sensory nerve ganglia and can later reactivate owing to waning cell-mediated immunity; this reactivation of latent VZV in a dermatomal distribution causes HZ (Gershon et al., 2015, Cohen, 2013). While varicella cases have dramatically declined with the introduction of the live-attenuated varicella vaccine, more than 100,000 annual cases of varicella continue to occur in the United States as of 2014 (Lopez et al., 2016). In addition, an estimated 1 million cases of HZ occur annually (Cohen, 2013). Meanwhile, more people than ever, across all ages and medical subspecialties, are receiving immunosuppressive medications or living with comorbidities that increase the risk for severe or complicated primary varicella infection in the absence of preexisting immunity (Moffat et al., 2007, Marin et al., 2013, Harpaz et al., 2016).

The purpose of this review is to provide an overview of the clinical presentation and epidemiology of VZV infections and to summarize post-exposure management guidelines in the United States. Providers who care for high-risk individuals (e.g., patients with cancer or on chemotherapy, transplant patients, patients on immunosuppressive medications for autoimmune/allergic diseases or severe skin conditions, pregnant women, or neonates) must educate patients to seek medical attention in the event of VZV exposure, be able to obtain appropriate assessment of immune status, and have rapid access to vaccination and immunoglobulin preparations.

## 2 Methods

Data included in this review article were identified by English language literature searches of electronic databases (MEDLINE, PubMed) through July 15, 2019 using the search terms “varicella-zoster virus,” “varicella,” “herpes zoster,” “transmission,” “epidemiology,” “at-risk populations,” “immunocompromised,” “vaccination,” “post-exposure prophylaxis,” “varicella zoster immune globulin,” and “VARIZIG.” Additional publications and reports were identified by searching the reference sections of relevant articles. Other data sources such as the Centers for Disease Control and Prevention were searched to access current vaccination rates, epidemiology reports, and guidelines and recommendations for clinical management of VZV infections.

This review was written as a narrative review to provide an overview of VZV clinical manifestations, epidemiology, potential complications, populations at increased risk, and current post-exposure management guidelines in the US.

## 3 Results and observations

### 3.1 VZV transmission

A highly contagious virus, VZV transmission primarily occurs two ways: 1) direct contact with skin lesions that are not fully dried and crusted (contact transmission); or 2) breathing in aerosolized viral particles from vesicular skin lesions (airborne transmission) (Gershon et al., 2015, American Academy of Pediatrics, 2018). The period of communicability extends from 1 to 2 days before rash onset until all lesions have crusted (Centers for Disease Control and Prevention, 2015). In unvaccinated individuals, clinical presentation of varicella typically includes 300 or more lesions and many vesicles (Lopez et al., 2018). In contrast, vaccinated individuals who develop varicella more than 42 days after vaccination (defined as breakthrough disease) typically experience a milder, shorter disease course characterized by fewer than 50 maculopapular lesions, with few or no vesicular lesions. Individuals who develop breakthrough varicella with no vesicular lesions should be considered infectious until no new maculopapular lesions appear within a 24-hour period (American Academy of Pediatrics, 2018). Secondary varicella infection rates range from 61% to 100% among susceptible household contacts exposed to patients with varicella (Gershon et al., 2018).

HZ is less contagious than varicella, but VZV transmission from patients with HZ does occur and can lead to development of varicella in susceptible persons (Cohen, 2013, Gershon et al., 2018). A surveillance study conducted in Philadelphia from 2003 to 2010 reported that in 9% of 290 HZ cases and 15% of 1358 varicella cases at least one other person became secondarily infected via VZV exposure (Viner et al., 2012). Individuals with HZ who had covered rashes on the trunk and those who had exposed rashes on the arms or hands were equally likely to spread VZV (Viner et al., 2012), consistent with another report of varicella cases in a long-term care facility in West Virginia originating with a resident with HZ whose trunk lesions were reportedly covered for the duration of the rash (Lopez et al., 2008). Other cases of VZV transmission to susceptible household members (Hoch et al., 2016) and health care workers (Josephson and Gombert, 1988, Saidel-Odes et al., 2010) in the absence of direct contact with skin lesions from individuals with localized HZ have been reported, but the extent to which lesions were covered is unclear.

### 3.2 Incidence of VZV infections in the vaccine era

#### 3.2.1 Varicella

In the US, incidence of VZV infection has decreased dramatically since the introduction of a varicella vaccine in 1995. Prior to the varicella vaccination program, approximately 4 million cases of varicella occurred annually, resulting in an estimated 10,000 hospitalizations and 100 deaths each year (Bialek et al., 2013). In the decade following implementation of routine 1-dose varicella vaccination in 1995, varicella incidence declined by 90%, hospitalizations by 88%, and deaths by more than 65% (Bialek et al., 2013). Despite high rates of vaccination, single-dose varicella vaccine effectiveness was approximately 85%, which was insufficient to prevent breakthrough varicella in immunized children or continued varicella outbreaks in schools and daycare centers (Seward et al., 2008, Shapiro et al., 2011).

In June 2006, the Advisory Committee on Immunization Practices recommended a routine second varicella vaccine dose (Marin et al., 2007). Effectiveness of 2 doses of the vaccine was 98% (Seward et al., 2008), resulting in additional declines in varicella incidence, from 25.4 cases per 100,000 population in 2005–2006 to 3.9 cases per 100,000 population in 2013–2014 (Lopez et al., 2016). Significant declines in varicella outpatient visits (60% decline) and varicella hospitalizations (38% decline) were also observed during the 2-dose varicella vaccination period from 2006 to 2012 (Leung and Harpaz, 2016). The annual varicella mortality rate during 2012–2016 was 0.03 per million population, representing a 94% decline from the prevaccine era and a 47% decline from the end of the 1-dose vaccination program (Leung and Marin, 2018). Implementation of routine 2-dose varicella vaccination was also associated with significant declines in the number of varicella outbreaks compared with the 1-dose era, as well as reductions in the size and duration of outbreaks (Bialek et al., 2013, Leung et al., 2015).

**Fig. 1:**
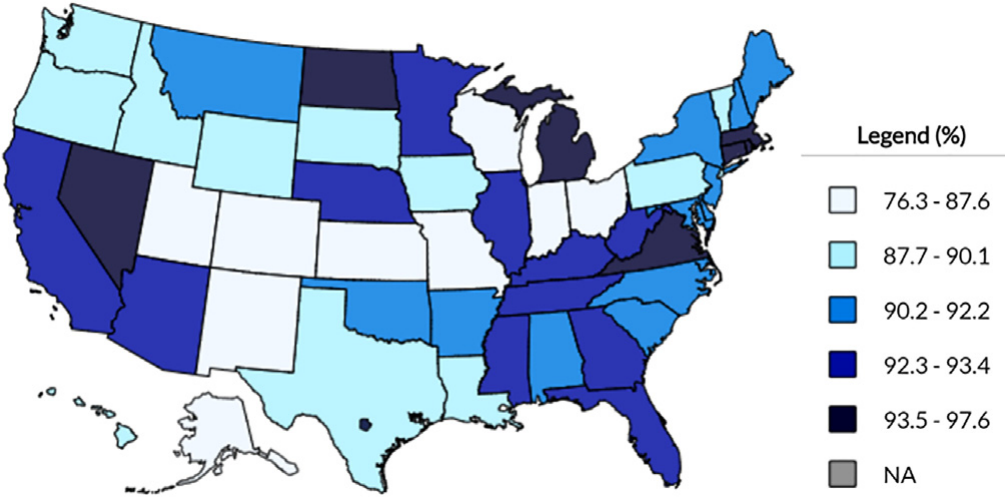
2017 Varicella Vaccination Rates (≥1 Dose) Among Children 19–35 Months of Age, by State, According to CDC Data from the National Immunization Survey-Child (NIS-Child) (Centers for Disease Control and Prevention, 2017a).

Maintaining high varicella vaccination rates is essential for preventing disease outbreaks and reducing the risk of VZV exposure (Lopez et al., 2019). As of 2017, rates of ≥1 dose of varicella vaccination among children 19–35 months of age in the US ranged from 76% to 98% (Fig. 1) (Centers for Disease Control and Prevention, 2017a). In addition to geographic disparities, vaccination rates differ amongst racial/ethnic groups and between foreign-born and US-born populations (Hill et al., 2018, Healy et al., 2018). Estimated 2-dose vaccination coverage rates needed to achieve population-level protection and prevent outbreaks range from 75% to 88% (Lopez et al., 2019). Between 2012 and 2015, 29 varicella outbreaks were reported in 10 sentinel jurisdictions with active varicella surveillance (Lopez et al., 2019). Undervaccination (i.e., no vaccination or 1-dose recipients) is associated with larger outbreaks (Lopez et al., 2019). Additionally, implementation of second-dose vaccination for outbreak control in 1-dose recipients reduced varicella incidence and severity (Nguyen et al., 2010). Together, this demonstrates the importance of a 2-dose varicella vaccination schedule. Increasing rates of undervaccination and vaccine refusal in recent years are associated with vaccine-preventable disease outbreaks (Phadke et al., 2016).

#### 3.2.2 Herpes zoster

Approximately 1 million cases of HZ occur annually in the US (Cohen, 2013). Two vaccines are approved by the FDA for prevention of HZ: 1) zoster vaccine live (ZOSTAVAX) was approved in 2006 (ZOSTAVAX Prescribing Information, 2006); and 2) zoster vaccine recombinant, adjuvanted (SHINGRIX) was approved in 2017 (SHINGRIX Prescribing Information, 2017). However, HZ vaccine uptake remains low; in the 2017 National Health Interview Survey (Centers for Disease Control and Prevention, 2017b), 35% of adults aged 60 years and older reported receiving HZ vaccination (primarily ZOSTAVAX, as SHINGRIX approval by the FDA and recommendation by the Advisory Committee on Immunization Practices occurred in October 2017 (Dooling et al., 2018).

Incidence of HZ among children in the US has declined approximately 70–80% since the introduction of the routine varicella vaccination program (Harpaz and Leung, 2019a, Weinmann et al., 2019). Population-based studies in the US have reported increasing rates of HZ among adults over the last 3 or more decades (Kawai et al., 2016, Harpaz and Leung, 2019b, Wolfson et al., 2019). For example, one study reported that HZ incidence among adults aged 35 years and older has increased from 2.5 cases per 1000 population in 1993, to 6.1 per 1000 in 2006 and 7.2 per 1000 in 2016 (Harpaz and Leung, 2019b). Although HZ incidence among adults has continued to increase, patterns vary by age strata, with recent plateaus among older adults (Harpaz and Leung, 2019b, Wolfson et al., 2019). The reasons underlying shifting patterns of HZ incidence among adults are unknown but appear unrelated to widespread varicella vaccination of children given that HZ incidence began increasing before introduction of varicella vaccine and did not increase more rapidly after routine immunization against varicella began (Hales et al., 2013, Kawai et al., 2016, Wolfson et al., 2019). In addition to rising rates of HZ at the overall population level, the incidence of HZ and frequency of complications such as postherpetic neuralgia are higher among immunocompromised subpopulations, per large insurance claims database studies in the US, UK, and Germany (Chen et al., 2014, Yanni et al., 2018, Schröder et al., 2017).

### 3.3 Populations at high risk for severe varicella infection and complications

Immunocompromised persons and other specific patient groups are at higher risk for severe varicella infections and complications (Table 1) (Centers for Disease Control and Prevention, 2015, Marin et al., 2013, Gnann, 2002, Moffat et al., 2007, Lamont et al., 2011, Bapat and Koren, 2013). Populations at higher risk for severe complications are also at higher risk for death (Moffat et al., 2007, Gershon et al., 2015, Gershon, 2017, Gershon et al., 2018).

**Table 1:** Populations at High Risk for Severe Varicella and Potential Complications (Centers for Disease Control and Prevention, 2015, Marin et al., 2013, Gnann, 2002, Moffat et al., 2007).

Populations at High Risk (Marin et al., 2013, Centers for Disease Control and Prevention, 2015)		
Immunocompromised patients without evidence of immunity Adults without evidence of immunity Pregnant women without evidence of immunity Newborn infants whose mothers have signs and symptoms of varicella around the time of delivery (i.e., 5 days before to 2 days after) Hospitalized premature infants born at ≥28 weeks of gestation whose mothers do not have evidence of immunity to varicella Hospitalized premature infants born at <28 weeks of gestation or who weigh ≤1000 g at birth, regardless of their mothers’ evidence of immunity to varicella		
Potential Complications	Example	Estimated Incidence Rate
Cutaneous (CDC, 2015; Gnann, 2002, Moffat et al., 2007)	Secondary bacterial infections of skin lesions caused by *Staphylococcus* or *Streptococcus* infections	Most common complication in children, causing hospitalization in 2–3 per 1000 cases Less common cutaneous complications include hemorrhagic varicella and purpura fulminans associated with thrombocytopenia and disseminated intravascular coagulation

Pulmonary (Gnann, 2002, Lamont et al., 2011)	Pneumonia	Radiographic evidence of varicella pneumonia is seen in 3% to 16% of adults Varicella pneumonia appears to be more severe and more frequent in pregnant women, complicating 10% to 20% of cases

Neurologic (Gnann, 2002, Moffat et al., 2007)	Cerebellar ataxia, encephalitis	Overall incidence of neurologic complications: 1–3 per 10,000 cases Cerebellar ataxia: 1 in 4000 cases Encephalitis: 1–2 episodes per 10,000 cases Rare neurologic complications include transverse myelitis, aseptic meningitis, optic neuritis, and Guillain-Barré syndrome

Congenital (Bapat and Koren, 2013)	Congenital varicella syndrome	1–2% of cases of maternal varicella during the first 20 weeks of pregnancy

#### 3.3.1 Immunocompromised population

The immunocompromised population is steadily increasing as a result of developments in medical management, new indications for immunosuppressive treatment, and greater life expectancy among immunosuppressed individuals (Harpaz et al., 2016). According to data from the 2013 National Health Interview Survey, 2.7% of US adults self-reported being immunosuppressed (Harpaz et al., 2016). This number may even be higher as the armamentarium of immunosuppressive agents continues to expand and is being increasingly utilized across a growing number of medical conditions and subspecialties (Harpaz et al., 2016, Wiseman, 2016, Bonura and Armstrong, 2017). For example, rates of transplantation have increased substantially over the past decade, according to the 2016 annual data report by the Organ Procurement and Transplantation Network and the Scientific Registry of Transplant Recipients (OPTN/SRTR, 2018). Kidney transplants—the most common solid organ transplant in the US—increased 7% between 2015 and 2016, from 18,597 to 19,859. Between 2007 and 2016, the number of liver, heart, and lung transplants has increased by 21%, 43%, and 56%, respectively. The annual number of hematopoietic cell transplantations reported in the US also continues to increase; according to data from the Center for International Blood and Marrow Transplant Research, more than 21,000 hematopoietic cell transplantations were performed in 2015, representing an approximately 60% increase over the past decade (D’Souza et al., 2017).

Cutaneous, pulmonary, and neurologic complications of varicella (Table 1) occur in both immunocompetent and immunocompromised individuals but tend to be more frequent in immunocompromised hosts (Gnann, 2002, Moffat et al., 2007, Gershon, 2017). Varicella infection is likely to be more severe and more prolonged in immunocompromised patients (Moffat et al., 2007, Gershon, 2017). Immunocompromised patients with varicella are particularly prone to develop pneumonia and hepatitis; these complications are associated with unchecked viral dissemination to the lungs and liver, respectively, and may be fatal (Moffat et al., 2007, Gershon, 2017). Certain immunosuppressive regimens may be associated with greater risk. For example, increased susceptibility to VZV infection (varicella or herpes zoster) has been reported in pediatric and adult solid organ transplant patients who received immunosuppressive regimens containing mycophenolate mofetil (Rothwell et al., 1999, Lauzurica et al., 2003, Gourishankar et al., 2004, Herrero et al., 2004, Koo et al., 2014, Hamaguchi et al., 2015).

#### 3.3.2 Pregnant women and newborns

VZV infection during pregnancy is associated with potentially serious complications, including maternal varicella pneumonia, congenital varicella syndrome, and neonatal varicella (Table 1) (Lamont et al., 2011, Bapat and Koren, 2013). Maternal varicella pneumonia is the most common complication of VZV infection during pregnancy. Approximately 10% to 20% of pregnant women acutely infected with varicella will develop pneumonia (Lamont et al., 2011, Bapat and Koren, 2013), and the risk for maternal varicella pneumonia increases between week 28 of pregnancy through delivery (Denny et al., 2018). Morbidity and mortality related to varicella pneumonia is higher among pregnant women than in the general adult population (Lamont et al., 2011, Bapat and Koren, 2013). Estimates of mortality related to varicella pneumonia approach 50% in pregnant women, compared with approximately 11% among healthy adults (Denny et al., 2018).

Congenital varicella syndrome is a complication related to maternal VZV infection occurring in the first 20 weeks of pregnancy (Lamont et al., 2011, Bapat and Koren, 2013). Clinical features of congenital varicella syndrome include fetal skin lesions, limb hypoplasia, neurologic abnormalities, and eye disorders. Prospective studies in North America and Europe suggest that congenital varicella syndrome occurs in 1% to 2% of infants born to mothers who had varicella infection in the first 20 weeks of pregnancy. More than 40 cases of congenital varicella syndrome are estimated in the US each year with a mortality rate of approximately 30% in the first few months of life (Lamont et al., 2011, Bapat and Koren, 2013).

Neonatal varicella infection can occur as a result of maternal varicella infection late in pregnancy, before passive immunity can be conferred from mother to baby (Lamont et al., 2011, Bapat and Koren, 2013). Acquisition of neonatal varicella occurs by one of three routes: 1) transplacental transmission; 2) ascending infection from lesions in the vaginal canal; or 3) the neonatal respiratory tract after birth. Transplacental transmission of VZV from mother to fetus is thought to be responsible for varicella infection occurring in neonates in the first 12 days of life, whereas postnatal VZV exposure is responsible for varicella infection in older neonates (between 12 and 28 days after birth). Neonatal varicella infection is associated with a low mortality rate overall, but risk of mortality is greater among premature babies (Lamont et al., 2011.

### 3.4 Clinical management of individuals exposed to VZV

Clinical management decisions should take into consideration evidence of immunity, type of exposure, and host-immune status with regard to ability to receive varicella vaccination safely. Methods to document varicella immunity are included in Table 2 (American Academy of Pediatrics, 2018, Marin et al., 2013). Recommended management of individuals exposed to someone with varicella or HZ is shown in Table 3 (American Academy of Pediatrics, 2018, Marin et al., 2013).

**Table 2:** Evidence of VZV Immunity (Marin et al., 2013, American Academy of Pediatrics, 2018, Lopez et al., 2018).

Methods Used to Verify VZV Immunity
Documentation of age-appropriate varicella vaccination Diagnosis or verification of a history of varicella or herpes zoster by a health care provider (parental or self-reporting is inadequate) Laboratory confirmation of disease (VZV PCR of skin lesions is the preferred method) Serum evidence of immunity (detection of IgG-class antibodies to VZV)^a^^b^ Birth in the United States before 1980 (should not be considered evidence of immunity for health care personnel, pregnant women, and immunocompromised persons)

IgG, immunoglobulin G; PCR, polymerase chain reaction; VZV, varicella-zoster virus.

a Serum specimens from individuals who have received blood products or intravenous or subcutaneous IgG in the past several months must be interpreted with caution, as the present exogenous IgG may confound detection of IgG-class antibodies to VZV (Bright et al., 2015).

b Serologic testing after vaccination is not recommended, as commercially available serologic assays are often insensitive for detecting vaccine-induced immunity (Lopez et al., 2018).

**Table 3:** Recommended VZV Post-Exposure Prophylaxis, by Immune Status (American Academy of Pediatrics, 2018, Marin et al., 2013).

Host	Recommended Prophylaxis	Alternative Prophylaxis	Timing of intervention^a^
Immune (evidence of immunity to varicella has been verified)	None indicated	None indicated	Any

No evidence of immunity to varicella Able to receive varicella vaccination	Varicella vaccination^c^	None indicated	Vaccination within 3–5 days post-exposure^b^, ^c^

No evidence of immunity to varicella At high risk for severe varicella Varicella vaccination is contraindicated	Varicella zoster immune globulin (VARIZIG®) 125 IU/10 kg body weight, up to a maximum of 625 IU, administered intramuscularly	If VARIZIG is not available: IVIG 400 mg/kg, preemptive oral acyclovir or valacyclovir	VARIZIG administration within 10 days (ideally within 96 h). If VARIZIG is not available, administration of IVIG should be considered up to 10 days post-exposure. If VARIZIG and IVIG are not available, a 7-day course of antiviral therapy beginning 7 to 10 days post-exposure may be considered.

IVIG, intravenous immune globulin; VZV, varicella-zoster virus.

a Recommended interval between VZV exposure and vaccination or passive immunoprophylaxis.

b Vaccination outside the 5-day window post-exposure still is recommended to help protect against potential future VZV exposures (American Academy of Pediatrics, 2018).

c The minimum interval between 2 doses of varicella vaccine is 3 months for children aged ≤12 years and 4 weeks for persons aged ≥13 years (American Academy of Pediatrics, 2018).

#### 3.4.1 Evidence of immunity

A number of options are considered appropriate criteria to verify immunity to varicella (Table 2) (American Academy of Pediatrics, 2018, Marin et al., 2013). One option to establish immunity is documentation of age-appropriate varicella vaccination: receipt of 1 dose for preschool-aged children (i.e., aged 12 months through 3 years) or receipt of 2 doses for school-aged children, adolescents, and adults. Secondly, a history of varicella or HZ diagnosed by a health care provider with either clinical examination or laboratory confirmation of disease (e.g., detection of VZV DNA by polymerase chain reaction in a skin lesion, respiratory tract specimen, or specimen from a sterile site) will establish immunity. Parental or self-reporting of varicella infection is considered inadequate verification of immunity. Serum evidence of immunity (e.g., detection of immunoglobulin G [IgG]-class antibodies to VZV) is often used as a criteria for evidence of immunity. However, interpretation of IgG-class antibodies in serum specimens from individuals who have received blood products or intravenous or subcutaneous IgG in the past several months must be done with caution, as the present exogenous IgG may make serum testing unreliable (Bright et al., 2015). Birth in the United States before 1980 is generally considered evidence of immunity but is inadequate for health care personnel, pregnant women, and immunocompromised persons. No post-exposure prophylaxis is needed in persons whose VZV immune status has been verified by one of these methods (American Academy of Pediatrics, 2018).

#### 3.4.2 Types of exposure

Types of exposure to VZV that are likely to result in infection in persons without immunity include household, playmate, and hospital exposures (American Academy of Pediatrics, 2018). Household exposure to VZV refers to exposure to an infected contact residing in the same household. Face-to-face indoor play of 5 min or more with an infected playmate is also considered significant exposure to VZV, although some experts suggest >1 h as the threshold for significant exposure through direct contact (American Academy of Pediatrics, 2018). Hospital exposure to varicella involves face-to-face contact with an infectious patient or staff member, a visit by a person deemed contagious, or sharing a room or adjacent beds in a large hospital ward with an infectious patient. Exposure to HZ is considered significant if there is close contact, such as touching or hugging, with a person deemed contagious (American Academy of Pediatrics, 2018, Marin et al., 2013). However, as noted above, VZV transmission from individuals with HZ can occur through either direct contact or airborne routes via inhalation of aerosolized virus from the rash (Josephson and Gombert, 1988, Lopez and Marin, 2008, Saidel-Odes et al., 2010, Viner et al., 2012, Hoch et al., 2016).

For individuals with varicella, airborne and contact infection control precautions should be implemented until lesions have crusted (Siegel et al., 2007), which usually takes at least 5 days after rash onset but often takes longer in immunocompromised patients (American Academy of Pediatrics, 2018). Individuals with uncomplicated varicella may return to school or work after vesicular lesions have crusted over, or, in cases of breakthrough infection with only macropapular lesions, until no new lesions appear within a 24-hour period (American Academy of Pediatrics, 2018). Infection control guidelines also include contact and airborne precautions for all patients with disseminated HZ and for immunocompromised patients with HZ until lesions have crusted (Siegel et al., 2007). Localized zoster lesions should be completely covered, if possible, until all lesions have crusted (American Academy of Pediatrics, 2018).

#### 3.4.3 Post-exposure prophylaxis in populations eligible for varicella vaccination

Varicella vaccine should be administered post-exposure to individuals who lack evidence of immunity to varicella, have no varicella vaccine contraindications, and are 12 months of age or older, including adults (Table 3) (American Academy of Pediatrics, 2018). Varicella vaccination within 3 days of exposure, and possibly up to 5 days, may prevent infection or mitigate disease severity (American Academy of Pediatrics, 2018). A Cochrane review of 3 randomized controlled trials of 110 healthy children who had household contact with infected siblings demonstrated the impact of varicella vaccination within 5 days post-exposure (Macartney et al., 2014). Significantly fewer vaccine recipients (23%) than placebo recipients (78%) developed varicella, and the majority of vaccine recipients who developed varicella had only mild disease. Although vaccination within 3 to 5 days of VZV exposure is recommended, risk of exposure may persist for weeks and even months in a varicella outbreak setting (Lopez and Marin, 2008). Thus, vaccination >5 days post-exposure is recommended to help protect against subsequent exposures and limit VZV transmission during an outbreak (Lopez and Marin, 2008, American Academy of Pediatrics, 2018).

#### 3.4.4 Post-exposure prophylaxis in populations with varicella vaccine contraindications

Varicella zoster immune globulin is recommended post-exposure for individuals at high risk for severe varicella disease who lack evidence of immunity to varicella and for whom varicella vaccine is contraindicated (Table 3) (Marin et al., 2013). In a varicella outbreak setting, active surveillance is recommended to identify high-risk individuals and implement appropriate control measures, including varicella zoster immune globulin (Lopez and Marin, 2008). Varicella zoster immune globulin recipients who become eligible for vaccination should receive varicella vaccination, as long as ≥5 months have elapsed since administration of varicella zoster immune globulin (Marin et al., 2013). In addition, it is recommended that household contacts without evidence of immunity receive varicella vaccination to reduce the risk of introducing wild-type VZV into the household (American Academy of Pediatrics, 2018).

Varicella zoster immune globulin is a purified immune globulin G preparation made from human plasma containing high levels of anti-VZV antibodies (VARIZIG Prescribing Information, 2018). High-risk groups who should receive varicella zoster immune globulin include those listed in Table 1. Administration of varicella zoster immune globulin is recommended as soon as possible following VZV exposure, ideally within 96 h but as late as 10 days post exposure (Marin et al., 2013). Consistent with these recommendations, an open-label study of VARIZIG in approximately 500 high-risk individuals exposed to varicella (263 immunocompromised adults and children, 137 pregnant women, and 105 infants) reported that post-exposure prophylaxis with VARIZIG was associated with low rates of varicella incidence when administered within 96 h (6.3%) or >96 h, up to 10 days (9.4%) (Levin et al., 2019). For high-risk individuals who have additional exposures to VZV ≥ 3 weeks after initial varicella zoster immune globulin administration, another dose of varicella zoster immune globulin should be considered (Marin et al., 2013).

Varicella zoster immune globulin can be obtained from a number of distributers beyond those listed in current guideline documents; a full list of options for obtaining varicella zoster immune globulin (VARIZIG) in the United States can be found at the following website: https://varizig.com/liquid-ordering_info.html (VARIZIG.com, 2018, accessed 17 July 2019). VARIZIG should not be confused with VZIG as this alternative varicella zoster immune globulin preparation is no longer commercially available in the United States. Providers in other countries in the world may need to contact their public health authorities for the best route to access varicella zoster immunoglobulin. VARIZIG has been obtained through patient/compassionate use channels in some countries when a shortage of registered product exists (S. Clement, Saol Therapeutics, personal communication, December 11, 2018).

If VARIZIG is not available, alternative interventions include intravenous immune globulin (IVIG) and antiviral therapy (American Academy of Pediatrics, 2018). Patients already receiving monthly high-dose (≥400 mg/kg) IVIG are likely to be protected and probably do not require varicella zoster immune globulin if the most recent dose of IVIG was administered 3 weeks or less before exposure (Marin et al., 2013). A 400-mg/kg dose of IVIG is sometimes used as an alternative to varicella zoster immune globulin (Table 3) (American Academy of Pediatrics, 2018). However, IVIG is not routinely tested for anti-VZV antibodies, and the titer in any specific lot of IVIG is uncertain (American Academy of Pediatrics, 2018). Moreover, clinical data supporting the effectiveness of IVIG for VZV post-exposure prophylaxis is lacking (American Academy of Pediatrics, 2018).

Some experts recommend 7 days of preemptive therapy with oral acyclovir (20 mg/kg per dose, administered 4 times per day, with a maximum daily dose of 3200 mg) or oral valacyclovir (if ≥3 months of age; 20 mg/kg per dose administered 3 times per day, with a maximum daily dose of 3 g) beginning 7 to 10 days after exposure (American Academy of Pediatrics, 2018). Preemptive oral acyclovir is sometimes used in addition to varicella zoster immune globulin or IVIG in the immunocompromised host (American Academy of Pediatrics, 2018). Preemptive oral acyclovir in VZV exposure has been studied only in normal healthy children. No studies have been conducted in adults or immunocompromised individuals (American Academy of Pediatrics, 2018).

No guidance exists in the literature for management of immunocompromised, non-immune patients who are receiving acyclovir or valacyclovir at prophylactic doses (e.g., during chemotherapy or after bone marrow or solid organ transplant) at the time of varicella or HZ exposure. While prophylactic antiviral therapy is often recommended to prevent VZV and herpes simplex virus reactivation (Tomblyn et al., 2009), it is unknown whether lower doses can prevent primary varicella infection in high-risk hosts. Given the risk for potentially devastating complications of varicella in these particularly susceptible hosts, the benefit of administering varicella zoster immune globulin to those without evidence of immunity despite use of prophylactic antiviral therapy may outweigh the risk until more data become available.

Future research is needed to evaluate the potential use of inactivated vaccines for prevention of varicella infection. One adjuvanted recombinant subunit zoster vaccine was approved in 2017 for prevention of HZ in adults 50 years of age and older (SHINGRIX Prescribing Information, 2017). Likewise, another inactivated VZV vaccine is in development that demonstrated efficacy in prevention of HZ and HZ-related complications in a phase 3 trial in autologous hematopoietic stem cell transplant recipients (Winston et al., 2018). Whether these inactivated vaccines can also prevent primary varicella infection in immunocompetent or immunocompromised adults will require additional studies.

## 4 Conclusions

Appropriate post-exposure management of individuals exposed to either varicella or herpes zoster should take into consideration the individual’s evidence of immunity, type of exposure, and host-immune status with regard to ability to receive varicella vaccination safely. Post-exposure prophylaxis with varicella vaccination in eligible immunocompetent hosts is recommended to prevent or mitigate infection, limit disease transmission, and help protect against potential future VZV exposure. Post-exposure prophylaxis with varicella zoster immune globulin is indicated for populations ineligible for vaccination, including immunocompromised children and adults, pregnant women, newborns of mothers with varicella shortly before or after delivery, and premature infants. In addition, vaccination of close household contacts is recommended to minimize introduction of VZV into households of immunocompromised persons who cannot be vaccinated. Providers who care for immunocompromised hosts should educate patients on the importance of urgently seeking medical care in the event of VZV exposure, particularly for patients known to have no evidence of immunity. Providers must be able to obtain appropriate tests to assess immune status and have rapid access to vaccination and varicella zoster immune globulin to avoid potential severe varicella complications.
